# How primary health care teams perceive the integration of oral health care into their practice: A qualitative study

**DOI:** 10.1371/journal.pone.0205465

**Published:** 2018-10-12

**Authors:** Hermina Harnagea, Lise Lamothe, Yves Couturier, Elham Emami

**Affiliations:** 1 School of Public Health, Université de Montréal, Québec, Canada; 2 Public Health Research Institute, Université de Montréal, Québec, Canada; 3 School of Social Work, Université de Sherbrooke, Québec, Canada; 4 Faculty of Dentistry, McGill University, Québec, Canada; University Lyon 1 Faculty of Dental Medicine, FRANCE

## Abstract

Recently, new models for the integration of oral health into primary care have been proposed. However, these models may be adopted by a variety of health care systems, and will reach successful outcomes only if they can be adapted to suit the local context. To this end, the objective of this study was to explore the perceptions of Quebec primary health care teams on the integration of oral health into primary care. A qualitative approach and interpretive description methodology were used to conduct the study within a case-study design. Purposeful sampling with maximum variation and snowball technique were used for recruitment of study participants. Seventy-four in-depth, semi-structured interviews and five focus groups were conducted with primary health care teams including health care providers and managers working in a rural and an urban health care center. The interview guide and study conceptual framework were based on the Rainbow model. Data collection and data analyses were conducted concurrently and continued until saturation was achieved. To analyze the data, four phases of qualitative analysis were followed. The thematic analysis included interview debriefing, transcript coding, data display, and interpretation. Data analysis was conducted both manually and with the use of Atlas-ti software. A total of four themes emerged from the interviews and focus group discussions. These themes covered all domains of the study theoretical model and included: 1) drivers of integration; 2) importance of integration; 3) professionals’ role in integrated care; and 4) barriers and enablers of integration. In general, most of the barriers expressed by study participants were related to the organizational and system domains of integration. Primary health care teams who provide care in rural and urban areas in Quebec expressed their concerns on the absence of integrated oral health services. Implementation of governance policies, the prioritization of educational and management measures as well as inter-professional collaboration toward innovative care models could facilitate this integration.

## Introduction

Various health organizations have proposed the integration of oral health into primary care as an effective approach to improve access to oral health care [1–4].

Primary oral health care has been defined by Isman [5] as “*the integration of services that promote and preserve oral health*, *prevent oral disease*, *injury and dysfunction and provide a regular source of care for acute and chronic oral diseases and disabilities*.” Although various demonstrations and pilot programs have shown promising results for the integration of oral health into primary care, the rate of implementation of this approach remains low [6–8]. Our comprehensive scoping review on this topic showed that one of the main barriers for primary oral health care is the lack of collaborative work and related suboptimal competencies of health care providers [9,10]. Accordingly, in many countries strategic health care plans have been implemented to promote interprofessional collaboration, increase knowledge, improve skills, and shape positive attitudes towards primary oral health care. As an example, the US Health Resources and Services Administration has encouraged primary health care providers to develop competencies in the areas of primary oral health care including: risk assessment, oral health evaluation, preventive intervention, counseling and education and interprofessionnal collaborative practices [11]. As defined by D'Amour and Oandasan [12], interprofessional work: “*The process by which professionals reflect on and develop ways of practicing that provide an integrated and cohesive answer to the needs of the client*, *family*, *and populations*.”

In fact, interprofessional collaboration has been introduced as the main facilitator for the integration of oral health into primary care. However, several recent studies conducted in Europe and North America have shown that many general practitioners and other non-dental health primary care workforce still have limited knowledge on interprofessional practice extended to the field of dentistry, and often have negative attitudes toward the adoption of primary oral health care in their regular practice [13, 14]. Characterized by teamwork and effective communication between various professionals, the collaborative practice could vary based on working environment, health care policies, and stakeholders’ perspectives, beliefs, and values in the real world of health care [15]. Understanding these values and identifying patterns among individuals’ perspectives are essential for planning and implementation of collaborative practices oriented toward primary oral health care as well as holistic global health care.

Therefore, the objective of this study was to answer the following research question: How do Quebec primary health care providers and managers perceive the integration of oral health into primary care and its associated barriers and facilitators?

## Methods

This study used qualitative research methodology within a case study design in a defined time and setting. Interpretive description, introduced by Thorne, was incorporated as a theoretical scaffolding [16]. This qualitative methodology has been selected as it generates useful findings in the context of clinical applied health care disciplines and it allows understanding the characteristics, patterns, and structure of a clinical phenomenon. In the interpretive description, the researchers’ foreknowledge of the studied phenomenon and in particular, their clinical expertise is considered to be the starting point for orienting the research design [16]. The inquiry can then be refined as the research progresses and finally, in the context of integrated oral health care, the research results will inform health care organizations to design and build an infrastructure that will facilitate this integration [16].

### Conceptual framework

The Rainbow model of Integrated Care introduced by Valentijn et al. [17] has been used as the study’s conceptual framework. This framework has been revised after reaching a consensus through an international Delphi process. According to this revised framework the integrated care can be extended from person-focus care to a well-defined population with specific needs [18] and on four levels: clinical, professional, organizational and system integration. The normative and functional integration has been identified as enablers of integration process.

### Study context and setting

In Canada dental care is mostly private thus costs are out-of-pocket or covered by employer-based insurance or via restricted specific programs for priority population. Such programs vary amongst province. In Quebec, publicly-funded dental care covers some priority population such as children under 10 years old, individuals covered by social insurance or those with special needs who receive hospital-based dental care. In 2015, in order to optimize quality, continuity, and accessibility of health care services, the Quebec Ministry of Health and Social Services changed the health care organizational structure by merging 182 health care organizations in different health regions into the creation of 34 integrated health and social services centers (HSSIC).

This research study was conducted in two public health care centers in the province of Quebec, Canada, from November 2016 to October 2017. The selection of study mega-centers (cases) was based mainly on their territories and population served. The first health care center serves an exclusively urban territory and offers services to a diverse population including a high number of seniors and new immigrants. The second health care center covers a larger territory including rural, semi-rural, and urban regions. In the latter center, the vast majority of the population consists of residents of the most disadvantaged rural region in Quebec. In both centers, oral health care services within the primary care sector were dispensed via the public health department under the supervision of a local public health dentist, offering free preventive oral health care services exclusively to children via center-affiliated dental hygienists.

### Participants’ selection

Purposive sampling, a maximum variation strategy, and snowball technique were used to identify and recruit study participants [19, 20]. Their eligibility criteria included being from the primary health care sector and having a least one year of work experience in this sector. The study participant recruitment continued until data saturation was reached. All identified individuals agreed to participate in the study.

### Data collection

Semi-structured interviews and focus group discussion were used as data collection methods. The focus groups included main teams of primary health care (public health, home care, children and youth health services) and were organized to stimulate the discussion between the study participants. Focus groups are particularly useful to explore shared visions and the degree of consensus on a specific topic [21]. The combination of two methods allowed gaining detailed, in-depth understanding of various professionals’ perspectives in addition to generating new insights from group reflection and a dynamic, real-world peer-to-peer interaction [22].

A preliminary interview guide (data in S1 File) with open-ended questions was developed by the research team (HH, YC, LL, EE), based on the Rainbow model and the existing literature on primary health care integration. The interview guide contained general questions on the role of health care providers in primary care, and specific questions related to oral health: population oral health needs, oral health evaluation processes, as well as clinical, organizational, professional, functional, normative, and systemic factors influencing oral health integration into primary care (data in S2–S5 Files). The interview guide was pilot-tested to ensure the clarity of questions, and necessary adjustments were made accordingly.

The research team has considerable experience in qualitative research and the doctoral candidate (HH) was trained in advance in qualitative research interviewing methods to collect the data. At the beginning of each interview and focus group, the interviewer (HH) introduced herself, provided information on the study, and explained the procedure. Then informed written consent was obtained from each study participant.

The audio-recorded face-to face (n = 68), telephonic interviews (n = 6) and focus group discussions (n = 5) lasted around 60–90 minutes and took place in the primary care sector of each health care center. The telephonic interviews were conducted to address some participants’ inability to participate in focus groups or face-to-face interviews because of time constraints.

Data collection and analysis were performed concurrently. This approach allowed us to expand the content of interviews and focus groups based on field-notes and ensured the reflexivity of researchers as well as validity of the saturation level [23, 24].

Ethical approval was obtained from the institutional review board of the Université de Montréal and health care centers.

### Data analysis

Following a thematic content analysis, four phases of qualitative analysis were identified as detailed by Bengtsson [23]: 1) decontextualization; 2) recontextualization; 3) categorization; and 4) compilation. Each of these stages was revised by other research team members who had deep knowledge on the integration concept in different disciplines. The involvement of an interdisciplinary research team prevented errors in data analysis, personal and interpretation bias, and ensured that no relevant data were excluded, thereby maintaining the confirmability and the credibility of the analysis [25].

In the phase of decontextualization, transcription of data was performed with repeated examination of recorded data by the first author (HH). Debriefing and reflexive field notes made it possible to better understand the context and the participants’ discourse. Then, an “inductive open coding process” followed [26]. The content areas were identified and accordingly, the text was divided into meaning units, which were subsequently condensed and transformed into codes. The use of a code explanation document facilitated the tracking of coding decisions, and maximized the dependability of the data analysis.

In the recontextualization phase, codes were reviewed against the original interview transcriptions. The research team regularly and thoroughly discussed uncertainties in coding and interpretation, and obtained agreement to ensure the study’s credibility.

In the phase of categorization, codes with similar conceptual content (meaning units) were grouped to identify the themes (Table 1).

**Table 1:** Example of a code tree.

Quotation	Open codes	Family	Category	Theme
**“We work a lot with mothers from the SIPPE program, we worked with fathers too, but we see a lot of mothers in prenatal classes … we do follow-ups for oral prevention.” (P1)**	Counseling and follow up—mothers and children +low income	Preventative needs—mothers and children +low income	Oral health needs—mothers and children +low income	Oral health needs as driver of integration
**“When I see that the tooth has an abscess, that it is swollen, […], I include oral hygiene in my plan.” (P3)**	Complicated caries Hygiene instructions (children)	Treatment for complicated caries (children) Preventative needs (children)	Oral health needs in children	
**“I see oral trauma, tooth loss, bleeding in the mouth […] avulsed teeth … yes, we see […] Alzheimer patients, they have food in the mouth, I see it between the teeth.” (P57)**	Dental trauma and bleeding (adults) Hygiene (cognitively impaired elderly)	Dental emergency treatment (adults) Preventative needs (cognitively impaired elderly)	Oral health needs in adults Oral health needs in cognitively impaired elderly	
**“Our young mothers think that the first teeth are not important, that we do not have to take care of them […] I have already seen a little baby a … little child at a given moment not even able to chew or to eat an apple because it's just … all his teeth were affected by bottle decay; so oral hygiene is not always well done. We had a person, we said!! the lady always has bad breath. This person wanted to eat more, but she couldn’t; we saw this poor lady, her mouth was full of abscesses […] Even for dentures you know? They don’t do it [hygiene] anymore …” (P28)**	Oral health education in young mothers ECC Hygiene (cognitively impaired elderly) Treatment complicated caries (elderly) Dental prosthesis hygiene	Preventative needs in mothers Caries treatment for children Preventative needs cognitively impaired elderly Dental treatment for elderly Prosthetic needs in elderly	Oral health needs in mothers Oral health needs in children Oral health needs in cognitively impaired elderly	
**“It could be trauma, it could be cancer.”(P33)**	Dental trauma Oral cancer	Dental emergency treatment Dental treatment (older persons)	Oral health needs (older persons)	
**“We still give the info, during the post-natal visit, you do not put the baby to sleep with the bottle, I give them advice for little things like that and what to put in the bottle too. A little basic information.” (P11)**	Information Education	Preventative needs (mothers)	Oral health needs (mothers)	
**“[…] poor hygiene, abscesses” (P29)**	Complicated caries Hygiene (children)	Treatment for complicated caries (children) Preventative needs (children)	Oral health needs in children	
**“Once I had 2 teenagers, both had really poor teeth, totaled […] it was obvious that … the little boys never smiled, they had bad breath coming out of their mouths, as a teenager you want girl friends, you want friends, and you smell very bad, who’d want to be friends with you?”(P5)**	Complicated caries (teens)	Treatment for complicated caries (teens) Preventative needs (teens)	Oral health needs (teens)	

Finally, the last stage (compilation) comprised analysis and interpretation. Although descriptive, the manifest analysis was followed by a latent analysis to understand hidden meanings of the original texts.

In the data interpretation, similarities and differences in rural and urban settings as well as the literature findings were considered, to maximize the transferability and authenticity of the research. Trustworthiness of the study was maximized by using principal guidelines in qualitative research [26]. A respondent validation was performed with 5 individuals.

Data analysis was conducted manually and was assisted by the use of Atlas-ti software (ATLAS-ti Scientific Software Development GmbH, Berlin, Germany).

Consolidated criteria for reporting qualitative research (COREQ) were followed.

## Results

Study participants (n = 91; men = 9; women = 82) included general practitioners (n = 9), nurses (n = 15), social workers (n = 21), managers (n = 14), educators (n = 10), nutritionists (n = 5), occupational therapists (n = 5), a speech therapist (n = 1), dental hygienists (n = 4), dental consultants (n = 2), and dentists (n = 5). The study participants’ work experience in primary health care varied from 1 to 32 years.

A total of four themes emerged from the interviews and focus group discussions and included: drivers of integration; importance of integration; professionals’ role in integrated care; and barriers and enablers of integration. These themes covered all domains and dimensions found in the study’s theoretical model: professional, organizational, clinical, functional, normative, and systemic; and at three levels: macro, meso, and micro level. Representative quotations illustrate how the interpretations were grounded in the data.

### Drivers of integration

This theme comprises two sub-themes as detailed below.

#### Oral health care service missing in publicly funded health services

The majority of study participants stated that, at the organizational and clinical level, integrated oral health services were either completely absent or largely insufficient in their primary health care organization:

"When our older patients need extractions, we are forced to refer them to hospitals […] it's very, very difficult and complicated. As [a doctor] I do not have support from a dentist. I find it very difficult to get this service."(P28)

"I think there is a major problem, there is a lack of service … it is inconceivable to see."()

"There is no program to address the condition of teeth, prostheses, these things, and then if it needs to be paid for, well … people do not go there"(P26)

#### Oral health needs as a driver of integration

Regarding the scope of an integrated care approach, both person-centered and population-centered needs were reported by study participants. For example, at the clinical level, for newborns and children, needs included parent’s lack of awareness and education on oral health, teeth/oral mucosa trauma and pain, oral-health-related problems in rare/genetic diseases, and lack of general anesthesia services for special needs children (intellectual or physical impairments).

“We are seeing children who refuse to eat, or children who have no weight gain …children who do not eat because they have toothache.”(P4)

“We need education, that’s really what we need […] One of the things I see here is that children are drinking a lot of pop, everybody drinks a lot of pop, most people drink a bottle of pepsi everyday… No, nobody drinks water here, doesn’t taste good apparently… So there has to be sugar in the water, and it’s better if it’s pop and it’s very hard to get them to go off it. So we’re dealing with … they get it at home, they get it very young, before they’re even in school, so maybe it has to start with the nurses … We have really good perinatal and early childhood nurses who see them for the vaccination, and they are the ones who work really close especially with these very underprivileged mothers.” (P18)

“We have certain children who need general anesthesia to remove caries, because they have intellectual impairments, they have autism or other disorders.”(P23)

Regarding elders, especially those staying in long-term health care facilities, the majority of health care providers highlighted an increase in the demand for integration of oral health into primary care because of an increase in the number of patients with multi-morbidities, impaired physical and mental health, as well as evolution in their oral health needs:

“At the beginning of my practice in 2002, most of the residents had dental prostheses; it was easy to clean, but now more people keep their teeth and the dental health is often bad, the teeth are missing, decayed, and in bad condition.”(P18)

“For sure, we need a dentist, we need a dentist because the people … the elderly … like today a day at minus 30 [degrees], if someone has a problem I am obliged to put them in an ambulance, or in an adapted transport, to send them here to the village.”(P9)

### Importance of integration

The non-dental health care providers, especially those being sensitive to health care challenges of vulnerable populations or tasked with patients’ oral health evaluation (e.g., nutritionists, occupational therapists, and nurses), were in agreement that the integration of oral health into primary care fits into the concept of holistic primary care and they considered it instrumental for the prevention of diseases and improving access to oral health care for specific populations:

“… it's the gateway to services … for all services” (P11)

“The mouth is important… When someone wants to express themselves … they are embarrassed, because there is bad breath or … because there is pain, caries …. the food … they don’t dare to smile, no.”(P19)

In the context of functional integration, they also highlighted the challenges of non-integrated care and its negative impact on general health:

“Well, generally I would not do that [evaluation and care of mouth], but I can realize the consequences of not doing so—either that the person is going to choke because she’s going to bed with food in her mouth, deterioration of the teeth will happen, and we may have an infection because there are cavities that settle in … after that there is an even more serious physical problem that will settle in.”(P18)

“There are some dentists… they refuse to take care of patients, especially those on welfare, for financial reasons, that's why for me it's a big problem … It's a problem we would not have if they were integrated into the health care network.” (P32)

Although the dentists participating in the study were not familiar with the concept of integration, they agreed that integration processes are necessary to improve the access to oral health care for vulnerable populations:

“…to be included in the RAMQ; personally, I had people who could not afford to pay for an extraction, then I think that in our society this should be a minimum. Minimum. If it is a tooth that has to be extracted because there is an infection, an apical lesion, periapical, pus that comes out everywhere…Even if it needs a root canal, if the person does not have the means, then she does not have the means … so minimally if it could be relieved by extracting the tooth. It happens! people who just … cannot afford to pay for an extraction … and that's … it's inhuman for me to say … ok, bye, take medicine, it'll hurt you again in two weeks…” (P77)

However, a few general practitioners emphasized that the problem of access to “needed care” will not be resolved by integration:

“I do not see the advantage of integrating these things. If you really want to have an advantage for the population, they should have access to dental and oral care, free of charge. In my region, which is very poor, if we really want to improve the oral health of the population, we should first improve access to these services, not integrate them … First of all.”(P31)

“I have the impression that it has to be public, it should be a public service, because if you want to do an integration it would have to be uniform for all the patients … and not only for some patients who have some ability to pay.”(P60)

### Professionals’ role in integrated care

In regard to professional integration, general health care providers positively perceived this integration and the majority considered oral health integrated care necessary to better accomplish their professional role. This role was described as participation in linkage and/or coordination processes.

" … there are vulnerable families, but sometimes you have to push, make the calls for them, because they do not have the instinct to call, make an appointment right now […] so we will try to push for them to be seen by a dentist.” (P16)

In the rural center, where the primary health care team included a dental hygienist, integration was reported as a coordination process including oral health screening to identify patients with special needs, sharing knowledge and clinical information as well as facilitating the transfer between settings:

"We have X [the hygienist] who interacts with us a lot, if there are things we do not know, we ask questions, we talk, we make visits with her, we refer.” (P16)

However, the majority of the health care providers believed that they didn’t have the necessary competencies to play an important role in oral health care and they highlighted the need of adding dental workforce to their team.

“I think I would have less work because at the moment any problem of the patient is referred to me, even though I am not the most competent person to evaluate it; any health problem so oral health problems too, even if my therapeutic arsenal is an antibiotic and a painkiller, it makes me think sincerely that, if there were a second, third line integrated, oral health problems would be referred directly to professionals more qualified than me, then I wouldn’t have to take care of this area, which is really not my specialty. I have the impression that if we had an integrated service it would be less work for me and not more.” (P24)

“In my point of view, the role of a doctor is mainly to cure the disease, it is especially the second line, it's not so much prevention. When you are really sick I think you need a doctor who knows what he is doing and how to do it. […] I have enough problems to be a good doctor to cure diseases; it's going to take me more time for something that should be done by public health…” (P26)

“It's true that it's a situation that sometimes puts us in funny positions, because it's not our specialty … then we can talk about parental skills, we can talk about …the ways of doing things, what if he was getting it done by his mother, you know? […] it's not necessarily my role and it becomes more delicate, but we … we talked about it … I recommended things, but I could not go too far either in this area …”(P52)

Some study participants considered that dental hygienist could play a role in primary health care because of their implication in preventive and public health approaches:

“…maybe a hygienist, because the young clientele is quite large.” (P59)

“I think, I see less the role of dentist … because we are in the primary care, prevention, … it would be more… I would see more a hygienist…” (P48)

“Well, I have the impression … tell me if I'm wrong … but it would be more to the level of dental hygienist you know? […] I think that our need, for the patients, would be more a dental hygienist in the sense that it is more awareness that we need… There are also serious cases, but it's less our daily life, I think it's more awareness and, hygiene.” (P49)

### Barriers and enablers of integration

Table 2 presents a summary of the barriers and facilitators and their representative quotations.

**Table 2:** Emergent themes of barriers and facilitators.

Domain/ Level	Barriers	Enablers
	*Themes*	
***Clinical integration*** ***(MICRO)***	**Fragmented care** *“They [dentists] are not a part of [the system]*… *when you need them to be there*, *you always have to go outside and it can take a lot of time*. *The fact that we have delays in these services… is because they are not* ***integrated*** *with us*, *are in another nucleus*, *completely separated*.*”(P27)* “… *so*, *at the hospital I do not think there are dentists doing surgeries*, *when we need to have our teeth extracted*, *which is often in the elderly*, *we have to go to hospitals in M*., *which offer general anesthesia service*, *and it's very*, *very difficult and complicated […] so that's a big*, *big problem*, *that we have no service on this side*.*”(P33)*	
***Professional integration*** **(MESO)**	**Suboptimal competencies** *“Patients with Parkinson’s*… *things like that*, *I have no idea what to do*, *how to treat them ” (dentist) (P67)* *“We will not at all be ready for it*, *we are not trained for that*. *The only things I remember learning in school are for example people who have a handicap*… *severe arthritis*, *well the tennis ball to help them then what*?… *We could continue to learn about these things*, *but I do not feel confident to act like that in front of a patient in his universe*… *Then he doesn’t want me to put my hand in his mouth and then he can be aggressive*… *this is too*… *it's too complex*, *delicate*, *we're not ready for that*.*”(P45)*	
***Organizational integration*** ***(*MESO)**	**Implementation challenges** *Subtheme*: ***Time constraints*** *“Then on top of the PAB work tasks we do already*, *taking on training on top of that*? *We are overwhelmed*.*”(P16)* *“Sometimes we are out of time too*, *as nurses*, *we’re on the road*, *you know*? *We see 5–6–7 sometimes 8 patients*, *that is*… *we are more*… *in the dental field we are more limited*, *we must stay on schedule*, *it is something that we leave aside*, *easily*.*”(P17)* *Subtheme*: ***Shortage of human resources and cost of services*** *“We need staff*, *time*, *we have to*, *right*? (…) *it involves all the staff*, *it involves quality staff*, *sufficient staff who*… *depending on the roles they play*, *nurses*… *more personnel*, *we are defective at this level*, *the number of personnel*, *we don’t have enough*, *and in sufficient numbers to give adequate care*… *”(P19)* *“We see the need*, *but we are short of manpower*… *We have no manpower to adequately meet the needs of the elderly*.*”(P18)*	**Care coordination mechanisms and interprofessional collaboration** *Subtheme*: ***Case management*** “Yes, it's always an interdisciplinary team and we all know each other. Then everyone has his/her own agenda and then let’s say… aaa… anything… after 7 weeks,… follows the 8th when the dental hygienist comes, and I inform the dental hygienist how it’s going… The baby is 9 weeks old, you can go, all that, then each person has his schedule and once it's done we fill in the intervention plan on file.”(P23) *Subtheme*: ***Interdisciplinary planning*** “… I think it's like a kind of intervention plan with all the different services, where, for example… the nurse, the school social worker and the dental hygienist will sit down then they will make an intervention plan in relation to the services, the needs of that client. I see it like that, integrating is where all the services are bound together in order to better help the child. ”(P7) “We saw that it works well, then we integrated it into our action plan, so we schedule an appointment… we notify J [the hygienist]… We then have to go together to visit the resident… the follow-ups work better with vulnerable residents especially."(P23) “If the hygienist notices disorders unrelated to teeth, she will draw my attention to them. Likewise, If I see something wrong with teeth I would notify her, we always worked well together.”(P22) *Subtheme*: ***Interdisciplinary education and training*** “I think that an aspect to be prioritized would be the training.”(P24) “We need education… this is what we need.” (P19) “We should have more staff, well trained staff. We agree that everything to do with nursing, everything evolves, like all other things, everything evolves, so we must be trained to keep up accordingly.”(P38) *Normative integration* *Subtheme*: ***Shared vision*** “All employees do prevention here. So, everyone is in primary care, but also with a prevention component… Because we said it's an important part.”(P23) *Subtheme*: ***Leadership*** “Is it allowed!?… We decided to implement it [hygienist as a team member] because we noticed… I'll tell you something, we implemented prenatal classes too. Because we decided this is an important part, the same thing with the nutritionist, then we looked at the development of the child, I am also a psycho educator, therefore, we looked at the development of the child then this is it, we decided it's important at a certain age, the appearance of the teeth, the baby bottle… anyway, plenty of such things, then we concluded that is the time when the hygienist should intervene.”(P23)
***System integration*** ***(*MACRO)**	**Oral health a low priority for health policy makers** *“I see [the DG]*, *then she is full of good will*, *but even if she decides in a year that she prioritizes BD care*, *then the ministry makes budget cuts*, *then other things happen*, *well*, *BD care is going to sit there then*, *isn’t it*? *That's it*, *it's politics*, *but it's the reality*.*”(P51)* *“If the management does not agree*, *it will not work*, *it is necessary that the management adheres also*.*”(P5)* *“Planning for dental care enrollment in institutions is not a priority at this time*.*”(P88)*	**Governance mechanisms** *Subtheme*: ***Supportive policies*** *“The other element that would be important is a legislative amendment for the representation of dentists in the decision-making bodies of institutions*. *To be more clear*, *the legislative changes that have been made at the level of the CMDP of QC don’t allow dentists to participate; there are seats reserved for the establishment’s Board of Directors for a representative of the medical specialists*, *there is a reserved seat for a representative of nurses*, *there is a reserved seat for a representative of GPs*, *can a dentist have access to one of these seats*?*”(P88)* *Subtheme*: ***Budget allocation and fundraising*** *“We always look in the community to see how we can proceed*, *but then it's case by case*, *right*? *To see what the difficulty is*, *is it financial*, *is it a transportation problem*, *or similar things*, *but we have partners in primary care as we have Transport Action*, *who can help the people*, *drive them*… *You pay a certain amount*, *then you are able to go to town or sometimes if it's medical*, *you can have it for free*, *so*, *we help with that and then we ask for subsidies from the population of*… *people in communities like the Lions Club*, *things like that*, *non-profit charitable organizations*, *or similarly businesses try to*… *do fundraising*, *we look at each case*, *what it needs and what we can do to help*.*”(P10)* “*I’ll answer you*… *there is money that can help*, *funding*.*”(P2)* “*I had 2 teenagers*, *for both of them their teeth were really bad*, *totaled*, *then anyway*… *everyone said you have to do something about that… anyway*, *I asked*, *I made requests for donations for them*, *I had their teeth extracted*, *we saw a dentist… and then we managed to do their teeth… after that I asked for donations to be able…*, *and so finally we gave back the smile to the children; it took me 3 years really*, *2 years and a half*, *3 years to succeed*, *to achieve that*.*” (P13)* *Subtheme*: ***Interorganizational agreements*** “*Like here*, *there is something new here*, *such as someone who falls*, *who is suspected of having a fracture*… *instead of sending them to the emergency room*, *we have the option at the CLSC to do it and the resident is coming back*, *so it could be something*… *a service corridor for the entire organization*, *I think a service corridor with a dental clinic could be very relevant there*.*”(P30)* “*Maybe there would be the creation of partnerships because dentists are often private practices*, *it's not like in the hospital where you can send someone*, *in case of an emergency*… *at the level of integration… it might be nice to have a partnership with a clinic that would be affiliated*.*”(P11)*
***Functional integration***		**Innovative models** “*You have to participate in the system*, *as part of pilot projects*, *and then allow for… let's say*… *more creativity*, *in modeling*, *to try different models and not be constrained in frameworks*, *mainly at the level of remuneration*. *To have creative frames*. *Thus to create models so that you get paid differently*.*”(P88)* Subtheme: **Guidelines and protocols development** “*But what I understand from what the team said*, *is that there is a need for tools*, *to equip themselves*, *to say when I*, *for example*, *me as SW*, *how do I address myself to the person to say*: *oh*, *my god*, *it stinks*… *We do not want to say that*, *because it undermines the bond of confidence that we have*. *Sometimes we have fairly complex files*, *but how can we prepare our employees so that they feel a little more comfortable*?*"(P70)* “*So*… *there will be patterns of communication*, *we could develop*… *general rules*, *in general*, *the patient*, *we could do this*, *we could do that*, *a kind of protocol we could implement to facilitate things*.*”(P21)* “*Clinical tools*, *protocols*, *transfer card*, *work plans*, *I do not know what a good oral assessment implies*, *it's once a week*, *a month*? *We*…, *I don’t have much time to take care of wounds*, *or feet*, *well*, *we'll just spend 10–15 minutes*, *we'll just*… *we'll get through*, *today it is oral health*, *because yes*… *oral health is important*.*”(P39)* *Subtheme***: *Co-location*** “*The fact of having him in the team*, *of being able to*… *let's suppose being able*… *physically*… *to be able to talk to him about cases*, *then to see a bit of his*… *I think that it helps a lot in terms of the confidence that we can have in this profession*, *in this staff*.*”(P70)* “*I do not know*… *maybe it would be working together physically in the same establishment*, *with a dentist*, *working together*, *then meetings to share each other’s concerns*, *and then an exchange*, *a communication and then an integration of what*… *we work together*.*”(P44)* *Subtheme***: *Developing performance indicators*** “*We go with perceptions*… *a deep belief*, *but we cannot measure the effectiveness of this type of professional*.*”(P45)* “*We already had some*… *other types of professionals*,… *you met them there*… *the lady*… *dental hygienists*… *in schools*, *it's difficult to assess the impact if*… *if I was asked if we need 5 more or so*… *if we can prove statistically*, *with*… *with numbers I do not know; say we went to around 6 to 10 residences*, *we noticed 60% or 80% have problems*. *and worse*, *20% major ones and this is something we should put in place*, *then there*… *we could ring a bell*.*”(P6)*

In general, most of the barriers expressed by study participants were related to the organizational and system domains of integration. These included the lack of health policies and governance mechanisms to facilitate the delivery of oral health services in the continuum of care. These gaps and other factors such as lack of competencies, shortage of qualified and trained primary care providers, and high costs of integrated services were most reported as barriers to primary oral health care. The most frequently reported facilitators were situated at the meso level and in fact represented the primary health care providers’ wishes for their health care organizations since the integration has not been realized in these organizations. These included the optimization of coordination mechanisms and interprofessional collaboration in the professional domain as well as supportive policies in the system integration domains, at the macro level. Other facilitators included budget allocation, fundraising strategies, and innovative care models.

## Discussion

This study explored the perceptions of primary health care teams on the integration of oral health into primary care. To our knowledge this is the first study conducted in Canada on this concept. Our results showed that in general, and regardless of geographical location of health care organizations, oral health care services were perceived as absent in the primary public health care sector and integration was seen as a vehicle to respond to the clientele’s oral health needs. The importance of oral health integration into primary care seemed to be rooted in the perceived oral health needs of priority population groups including children, adolescents, and elders, and was linked to the providers’ incapacity to address these needs in the current health care system in Canada. Furthermore, primary health care teams both in rural and urban health care centers reported an association between the oral health needs of the priority population and their professional roles. These results are in line with the findings of previous studies [27] that demonstrated the positive impact of perceived professional responsibility on integration processes and collaborative practices. In our study, these collaborative practices were mostly expressed in terms of linkages and coordination processes. Furthermore, although the lack of primary oral health care services and the importance of oral health care integration were highlighted by the study participants, they did not have concrete ideas on the mechanism of integration into their current practice. Thus, for some general practitioners, specially those working in rural areas, the concept of integration was not considered as an element of public health as long as services are not free of charge. These issues can be explained by the fact that primary care providers understand the clinical operational processes of integrated care better than theoretical models, taxonomies, and parameters of integrated care. Furthermore, dental and oral health care is considered mostly as a secondary line of care provided solely by dentists. Several previous studies [28, 29] concluded that oral health promotion and prevention of oral diseases must be “everybody’s business.” Our study adds to these findings that the progress of oral health integration into primary care necessitates tailored designs and role-specific approaches which take into account each profession’s needs in practice, analyzing both context and group interrelations. Professionals’ age did not appear to be a contributing factor in integration. In our study older and more experienced professionals expressed generally the same views as younger ones. On the other hand, rurality seemed to empower positive perception on the integration via interprofessional collaboration. This could be explained by the oral health needs that vulnerable populations encounter in rural and remote areas.

There was largely a consensus across health care providers of various disciplines on the types of barriers and facilitators in terms of primary oral health care, but the importance accorded to these varied from one discipline to another. For example, at the micro level, in the clinical integration domain, in silo practices was a barrier reported mostly by general practitioners and dentists, leading to fragmented care and absence of structured coordination mechanisms. There is a global knowledge that oral health is part of general health and thus exclusion of dental care from primary care can lead to major harms to patients’ health [30, 31]. Despite various initiatives intended to close the gap between medical and dental care through integrated health care programs, lack of coverage of dental care by medical insurance and the limited number of dentists working in the public sector remain major problems to address in meeting the oral health needs of priority populations [32].

Our study results are in line with previous studies that recommend the development of core competencies for primary care providers as the strategy to address this barrier [9, 11]. Increasing the education and training of primary care practitioners will lead to early detection of oral diseases and will empower preventive approaches [27]. Some participants raised aspects of ageism, age discrimination in providing care for elders, especially when prevention is of interest. This could be related to several issues such as limited empirical evidence for treatment of chronic diseases, health professionals’ view of aging as a process of decline, and preferences for using their knowledge and skills to cure acute illnesses rather than managing chronic conditions.

Some studies have shown that practice integration could be facilitated through interventions that don’t require additional resources [33]. Structured protocols and the history of collaboration of primary care teams with dental hygienists through public health programs in Quebec seem to have contributed to oral health awareness among the primary care providers. Therefore, the integration of oral health care into primary care teams could be realized by the addition of allied dental workforce to the primary care teams without adding substantial cost to the health care system.

In regard to the organizational integration, time constraints and roles ambiguity seem to be important barriers in the provision of oral health care by members of health care teams such as nurses and social workers. However in terms of functional integration, limited interoperability of information systems, lack of appropriate tools and guidelines, as well as various logistical aspects such as dentists’ remuneration policies or dental equipment availability were reported by study participants as demotivating factors in adopting integration processes in interdisciplinary teams. At the macro level and in the systemic domain, lack of human resources, integrated services costs, and low political priority accorded to oral health were reported mostly by managers. These findings were similar to results of our recent scoping review [10] on the integration of oral health into primary care.

Several limitations should be noted for this study. First, our study didn’t include the perspectives of patients in regard to the integration of oral health into primary care. Secondly, our results cannot be considered as exhaustive due to their sensitivity to the teams and context. For example, the composition of primary healthcare providers in the public health care system in Quebec is skewed towards females, which was heavily reflected in our sampling. Views and perceptions on oral health integration into primary care could be related to gender differences. Likewise, the transferability of the findings to other contexts or settings, which is equivalent to external validity in quantitative research, could be considered as a study limitation. However, we enhanced the transferability of findings by providing a detailed description of the specific context and assumptions that were central to the research. Finally, like any qualitative study, the researcher was the primary instrument for data collection and analysis. Despite this inevitable subjectivity of the study, considerable overlap of some thematic content across health care disciplines suggests that there is a shared understanding of integration aspects and that our findings might be transferable to other settings. Further studies are needed to explore the extent of providers’ views and to address the various levels of integration in order to deliver oral health care in the primary care sector.

## Conclusion

Primary health care teams who provide care in rural and urban areas of Quebec expressed their concerns on the absence of integrated oral health services. Our study results suggest that advancing oral health integration into primary care necessitates adjusted designs and role-specific approaches which take into account each profession’s needs in practice, analyzing both context and group interrelations

Implementation of governance policies, the prioritization of educational and management measures as well as inter-professional collaboration toward innovative care models could facilitate this integration.

## Supporting information

S1 File Preliminary interview guide. (DOC)

S2 File Interview guide: Non-dental providers. (DOC)

S3 File Interview guide: Dentists. (DOC)

S4 File Interview guide: Managers. (DOC)

S5 File Focus groups guide. (DOCX)
