# Supplementary material for: How primary health care teams perceive the integration of oral health care into their practice: A qualitative study
Source: PLoS One. 2018 Oct 12;13(10):e0205465. doi: 10.1371/journal.pone.0205465 (PMC6185726; doi:10.1371/journal.pone.0205465)
Supplement: S2 File — (DOC) [file pone.0205465.s002.doc]

| **InterviEW plaN** |
| --- |

Date: …………………………..

Number of interview: ………………………….

Time and place:

1. **Opening the Interview**

- Greet the applicant; introduce yourself (interviewer /position) and make the interviewee feel comfortable
- Explain the purpose of the interview, inform candidate of the duration of the interview allow interviewee to read
- Invite the interviewee to clarify any doubts regarding the interview, and sign the 2 copies of consent form (keep 1 copy)
- Describe the interview process
- Before starting mention to participant that :

Your anonymity will be respected:

“Your name will appear on no report, on no published document. If you quote names of persons during the interview, these names will not be revealed”

- Underline that: "even if there is nothing really very indiscreet in the discussion, you can refuse at any time to answer or still to end the interview "
- Install the tape recorder (make test of recording)

1. **CONDUCTING THE INTERVIEW**

- Begin with unstructured, open questions to encourage the spontaneity, then encircle gradually the subject (funnelling technique)
- Make brief syntheses at the end of sections to make sure that we understood well and to give the opportunity to individual to complete or to modify its statements (reformulation: " if I understood well what you say to me, you ….")
- It is possible that, spontaneously, individuals approaches the themes of discussion on a different order which that describes in the following sections (for example in passing directly of the section 1 in the section 3): in that case, not to try to respect the order of sections, the important is to facilitate its spontaneity while making sure that we approach all the strategic themes.

**SECTION 1: BACKGROUND**

1. What kind of role do you play among the team of professionals at this organisation / facility? And how many years have you spent in this capacity?) How about before that? Have you always been involved in similar roles ?)
2. What is your definition for primary care?
3. What is your motivation for working in the primary care?

**SECTION 2: CLINICAL IntegrAtioN (PRESENT or ABSENT)**

1. In your daily experience what are the clientele that you work for? What would you say are the most common needs / care requirements of the majority of your clientele on a day to day basis?
2. Let’s say that I am a patient who comes to see you for a reason X, how do you proceed to evaluate me? (Do you have any protocols).
3. Do you consider oral health in your evaluation?
4. Which are the difficulties in oral health evaluation
5. Let’s say I’m a patient with a tooth ache what would you do with this information?
6. What role do you play in oral health?
7. What kind oral care could you provide in primary care?
8. What are the elements (personal, professional, organizational) that may help you to contribute to a better management of oral health?
9. When you identify an oral problem that you cannot handle what do you do?

**SECTION 3: The need OF INTEGRATION**

1. What is for you service integration?
2. Do you see any difference between coordination and integration?
3. When you hear about integrating oral health at primary care level what comes to your mind?
4. How do you think integration would benefit the population ( oral health and access)
5. What resources are available in your organization to integrate oral health care in the primary care?
6. What do you think about the role of different members of primary care ( nurses, long-term services) How do you think managers could play a role towards achieving integration?
7. Who should be involved? How do you think each partner will be able to contribute their opinions?
8. In your centre do you say the oral health services are integrated in primary care?
9. In your job capacity have you been familiar with or involved in any initiatives or discussions on integration of oral services at PHC level? I would like to know more about your experience – could you tell me how did you come about to be involved in such initiatives? What was the outcome?
10. What would be an opposite situation, with no integration?
11. What do we need to change in the organization

-clinical information systems

-clinical tools

-coordination tools

-clinical guides

1. How do you think the process should be initiated…practically speaking where do we begin? leadership
2. How do you think your experience with dental services would be if the services were integrated?

**SECTION 4: ORGANIZATIONAL IntegRation**

1. How can the organization help in better management of oral health?
2. What are the facilitators and barriers (organizational) with regard to integration of oral health in primary care?
3. How does your current position allow you to play a role in the integration of oral health in primary care?
4. How do you think integrated care would be received (have been received) by professionals, manager and policy makers?

**SECTION 5: Normative IntegRation**

1. How do you feel about one single universal model of oral health care integration would address the needs in all kinds of community settings?
2. How we can develop norms, practical guidelines, protocols for other health care professionals to further achieve the integration of oral health care?

**SECTION 6: IMPLEMENTATION**

What kind oral care would you wish be provided in primary care? (in which section: long-term care, schools,….)

1. **Closing THE INTERVIEW**

- Is there anything else which seems to you important and that you would like to talk about? any comment?
- Thank candidate for their time and contribution
- Ask if the participant can be contacted later if necessary

**D) AFTER THE INTERVIEW**

- Verify if the tape recorder
- Write down any observations made during the interview
