# Supplementary material for: How primary health care teams perceive the integration of oral health care into their practice: A qualitative study
Source: PLoS One. 2018 Oct 12;13(10):e0205465. doi: 10.1371/journal.pone.0205465 (PMC6185726; doi:10.1371/journal.pone.0205465)
Supplement: S5 File — (DOCX) [file pone.0205465.s005.docx]

To be addressed:

- purpose of the session

- invitation to express oneself freely

- rules of participation

- confidentiality

Introduction to the discussion - (5 minutes)

Hello everyone

My name is H and first, I want to thank you for your interest in this project which aims to describe the way you perceive the integration of oral services into your daily practice.

In this interview, I essentially want to know your opinion about your role in the oral health of individuals. The exchanges will last about an hour.

There are no good or bad answers, only different points of view. Neither is it necessary to reach a consensus. Everyone should feel comfortable expressing their opinion.

To facilitate the discussion, I ask you to speak loud enough for everyone to hear, and only one person at a time. The discussion is recorded because I do not want to lose any of the opinions expressed. During the discussion, I will only use your first names if necessary, but in my report these will be replaced by your function and numbers, to preserve anonymity.

My role is to ask questions and listen. I will not participate in the discussions. I invite you to talk to each other instead. I will take notes on the discussion.

In group discussions, some tend to speak more than others. As I want to hear from everyone because each of you has a special experience, it is possible that I will interrupt the person speaking and invite others to express themselves more. I also invite you to take notes on the sheets available to you so as not to lose your idea while waiting for your turn.

Are there any questions about the course of the meeting?

Now, I invite you to return the consent form distributed before the beginning of the meeting.

Question 1

5 minutes

What does integrated health care services mean to you?

Question 2

7-10 minutes

If you had to describe your role in relation to oral health, how would you describe this role?

What types of services / oral care can you offer?

Question 3

10 minutes

What are the challenges in fulfilling this role?

In your opinion, how could we overcome these challenges?

Question 4

5 minutes

Brief round table

What does integration of oral services mean to you?

Question 5

20 minutes

How can this integration be achieved / What should be done?

Clinical information system (e.g., files), clinical tools, assessment tools, planning tools, coordination mechanisms such as case management or outreach worker, interprofessional and cross-sectoral protocols.

Concluding focus group

3-5 minutes

Has something been forgotten? Do any of you wish to add anything else, to clarify?

I invite participants who feel that they have not been able to say everything they wanted to during the exchanges that have just taken place, and who would like to provide their view in writing, to send their thoughts by email to the address indicated on your information form.

We sincerely thank you for your participation in this research. The results of the focus group analysis will be made available to you later.
